# Human Fibroblasts as a Model for the Study of Bone Disorders

**DOI:** 10.3389/fendo.2020.00394

**Published:** 2020-06-19

**Authors:** Lauria Claeys, Nathalie Bravenboer, Elisabeth M. W. Eekhoff, Dimitra Micha

**Affiliations:** ^1^Department of Clinical Genetics, Amsterdam Movement Sciences, Amsterdam UMC, Vrije Universiteit Amsterdam, Amsterdam, Netherlands; ^2^Department of Clinical Chemistry, Amsterdam Movement Sciences, Amsterdam UMC, Vrije Universiteit Amsterdam, Amsterdam, Netherlands; ^3^Department of Internal Medicine Section Endocrinology, Amsterdam Movement Sciences, Amsterdam UMC, Vrije Universiteit Amsterdam, Amsterdam, Netherlands

**Keywords:** fibroblast, preclinical model, osteoblast, bone disease, osteogenic transdifferentiation

## Abstract

Bone tissue degeneration is an urgent clinical issue, making it a subject of intensive research. Chronic skeletal disease forms can be prevalent, such as the age-related osteoporosis, or rare, in the form of monogenetic bone disorders. A barrier in the understanding of the underlying pathological process is the lack of accessibility to relevant material. For this reason, cells of non-bone tissue are emerging as a suitable alternative for models of bone biology. Fibroblasts are highly suitable for this application; they populate accessible anatomical locations, such as the skin tissue. Reports suggesting their utility in preclinical models for the study of skeletal diseases are increasingly becoming available. The majority of these are based on the generation of an intermediate stem cell type, the induced pluripotent stem cells, which are subsequently directed to the osteogenic cell lineage. This intermediate stage is circumvented in transdifferentiation, the process regulating the direct conversion of fibroblasts to osteogenic cells, which is currently not well-explored. With this mini review, we aimed to give an overview of existing osteogenic transdifferentiation models and to inform about their applications in bone biology models.

## Introduction

Bone disorders encompass a wide range of chronic disorders with diverse etiologies, including both genetic and environmental factors. Next generation sequencing has contributed to the identification of the responsible genomic loci for several of the 500 Mendelian bone disorders, which has expanded our understanding of bone biology and its pathology in more frequent conditions, such as fractures at a postmenopausal age (1, 2). Nonetheless, for many of these patients this information remains to be translated into meaningful therapies. This gap between the genetic breakthrough and treatment can be largely attributed to the lack of cell models relevant for the study of bone tissue, in which the (genetic) defect can be examined and interrogated for the exploration of a therapeutic intervention. Such a model also offers the prospect of screening the large number of genetic variants of questionable pathogenicity, which are frequently found in exome analyses (3).

The paucity of bone material, which can be understandably explained by the invasive nature of the biopsy, has prompted efforts for the invention of bone cell models, which can faithfully recapitulate the disease mechanism by making use of more accessible patient materials. In the recent years, the attention toward fibroblasts as a starting point for differentiation to other cell types, including osteoblasts, has been growing. Fibroblasts are a common resident cell type in connective tissue found almost ubiquitously in the human body, including the easily accessible skin. Despite their recognition as a prominent cell type for about a century (4), their characterization remains obscure due to the lack of suitable markers and their uncharacterized diversity (5). However, their potential in osteogenesis can be demonstrated both in nature, in disorders of pathological ossification, as well as in more artificial systems of *in vitro* osteogenic differentiation. This allows the consideration of fibroblast-based models for the study of bone disorders. Particularly, this review focuses on human fibroblast-based models of osteogenic transdifferentiation for modeling of osteoblast-dependent disorders (Table 1).

**Table 1:** Overview of osteoblast cell derivation approaches.

**Fibroblast**	**Approach**	**Mouse model**	**Reference**
Gingival, dermal	Retroviral delivery of RUNX-2, Osterix, Oct3/4 and L-Myc in combination with ascorbic acid, β-glycerophosphate, dexamethasone.	NOD/SCID	(6)
Dermal	Retroviral delivery of Oct9 with N-Myc in combination with ascorbic acid, β-glycerophosphate, dexamethasone.	-	(7)
Gingival, dermal	Plasmid delivery of Oct4, Osterix, and L-Myc in combination with ascorbic acid, β-glycerophosphate, dexamethasone.	NOD/SCID	(8)
Gingival, dermal foreskin	Adenovirus delivery of BMP7.	NIH III, C57BL/6	(9–11)
Gingival, dermal foreskin	Ascorbic acid, β-glycerophosphate, dexamethasone.	–	(12)
Dermal	Ascorbic acid, β-glycerophosphate, human platelet lysate.	–	(13, 14)
Gingival	Ascorbic acid, β-glycerophosphate.	–	(15)
Interspinous ligament	Osteoclast cell-like conditioned media.	–	(16)
Dermal	Ascorbic acid, β-glycerophosphate, dexamethasone, ALK5 inhibitor II, vitamin D.	NOG	(17)
Dermal	Ascorbic acid, β-glycerophosphate, dexamethasone, TGF-β.	–	(18)
Dermal	Ascorbic acid, β-glycerophosphate, vitamin D, p-tricalcium phosphate scaffold.	–	(19)
Gingival	5-aza-dC and BMP-2.	BNX	(20)

## Bone Biology

In order to appreciate the value and shortcomings of relevant cell models, an overview of bone biology is required. The bone tissue typically consists of the mineral and organic components, which confer its stiffness and flexibility, respectively, to ensure its competence during continuous exposure to mechanical stress. Particularly, collagen type I constitutes the largest part of the organic mass; in addition to supporting cell growth and function, it also serves as a scaffold for mineral deposition. Bone tissue adapts to environmental cues by constant remodeling, which is primarily orchestrated by three different cell types, the osteoblasts, osteoclasts, and osteocytes. Osteoblasts have an anabolic role in building bone tissue by secreting the collagen-rich extracellular matrix (ECM) whereas osteoclasts perform a catabolic function by degrading the bone tissue. In this setting some of the osteoblasts become buried in the mineralized ECM, which triggers their differentiation to osteocytes. The latter are mechanosensing cells, which coordinate the function and differentiation of osteoblasts and osteoclasts, depending on their exposure to mechanical loading (21). Considering the closely interconnected relation of these cells, it is easy to deduce that defects in osteoblast differentiation or function can cause disease by influencing the net effect on bone mass development (22, 23). Thus, models allowing the study of osteoblast biology can be insightful in delineating the disease mechanism.

## The Humble Fibroblast

The fibroblast is generally known as a spindle-shaped adherent cell type and a common resident of mesenchymal stroma. It has been considered as a rather inert cell type for many years with the sole role of producing large amounts of ECM proteins, such as collagen type I, intended for the homeostasis of the connective tissue. However, it is steadily becoming more clear that fibroblasts have a much broader function, which includes the regulation of immune and inflammatory responses, for example during cutaneous wound repair (24), as well as during cell differentiation and behavior of neighboring cell types (25). It is also accepted that they represent a heterogeneous cell population, whose diversity extends not only across different anatomical locations but also within the same tissue, such as in the skin layers (26). Despite their abundance, their precise nature remains poorly characterized since they lack specific defining markers. It is perhaps partially because of this unspecialized character that they exhibit such a great plasticity and the ability to differentiate into other somatic cell types including osteogenic cells (27). Interestingly, it is a topic of great discussion whether fibroblasts are in fact the same cell type as mesenchymal stem cells (MSCs). According to the guidelines of the International Society for Cellular Therapy they are, as both cell types are plastic-adherent, share the presence and absence of the same MSC markers, and can differentiate into cells of the osteogenic, adipogenic, and chondrogenic lineages (27–29). MSCs were originally isolated from the bone marrow but have been subsequently identified in many tissues, including the skin. Their osteogenic properties have raised scientific attention with regards to their application in regenerative medicine (30–32). The similarities they share with the bone-forming MSC progenitors support the use of fibroblasts as an appropriate cell type to study osteogenesis.

## Fibroblast-Based Models for the Study of Bone Disorders

In the recent years, a plethora of reports have emerged, exploring the osteogenic properties of fibroblasts in producing osteoblasts suitable as disease models, as well as for potential bone regenerative applications. These refer mainly to two different ways of cell reprogramming for derivation of osteoblast cells: induced pluripotent stem cell (iPSC)-mediated differentiation and transdifferentiation. The first is based on the dedifferentiation of fibroblasts to an artificial stem cell type (iPSCs) by the induction of pluripotency. It is accomplished by the forced expression of the “Yamanaka factors” which typically include the Oct3/4, Sox2, c-Myc, and Klf4 transcription factors (33, 34). The iPSCs can be then directed toward the osteogenic cell lineage. The excitement revolving around the promising results of this approach is undoubtedly reflected in the numerous ongoing studies (35, 36). In addition to the multipotent plasticity of these cells, their patient specificity for autologous treatment and the lack of associated ethical issues, iPSCs have emerged in the last decade as a source of induced MSCs (iMSCs) (37), which are reported to have superior qualities to those of primary MSCs in cell survival and engraftment (38–40).

Despite these advantages, it is widely acknowledged that there are certain considerations with the use of iPSCs, such as the requirement for specialized technical resources for reprogramming and the consequences of manipulation for the induction of pluripotency, which include teratoma formation in regenerative applications. Minimization of these risks could be accomplished by optimizing the delivery of pluripotency factors by switching to non-integrative approaches, ensuring the absence of residual undifferentiated iPSCs, and monitoring the off-target effects (41). Another point of consideration is the potential disturbance of the cells differentiation potential as a result of reprogramming. Thus, iPSCs may not be suited to study a disorder in which the defect lies in cell differentiation. This is exemplified by the inhibition of iPSC generation and maintenance from fibroblasts of patients with fibrodysplasia ossificans progressiva (FOP), a severe disorder of heterotopic ossification. This was reported to be caused by the gene defect of the disease in the activin receptor-like kinase 2 (*ALK2*) gene (42). These problems have been resolved in studies, in which iPSCs, and iMSCs from patient fibroblast-derived iPSCs, have been successfully used in FOP disease modeling (43, 44). Perhaps partly owing to these limitations, a low number of studies exist, concerning iPSCs from patients with rare bone disorders. In addition to FOP, these include iPSCs from Marfan syndrome fibroblasts (45), osteogenesis imperfecta bone marrow MSCs (38), craniometaphyseal dysplasia peripheral blood cells (46), thanatophoric dysplasia, and achondroplasia (47).

**Figure 1:**
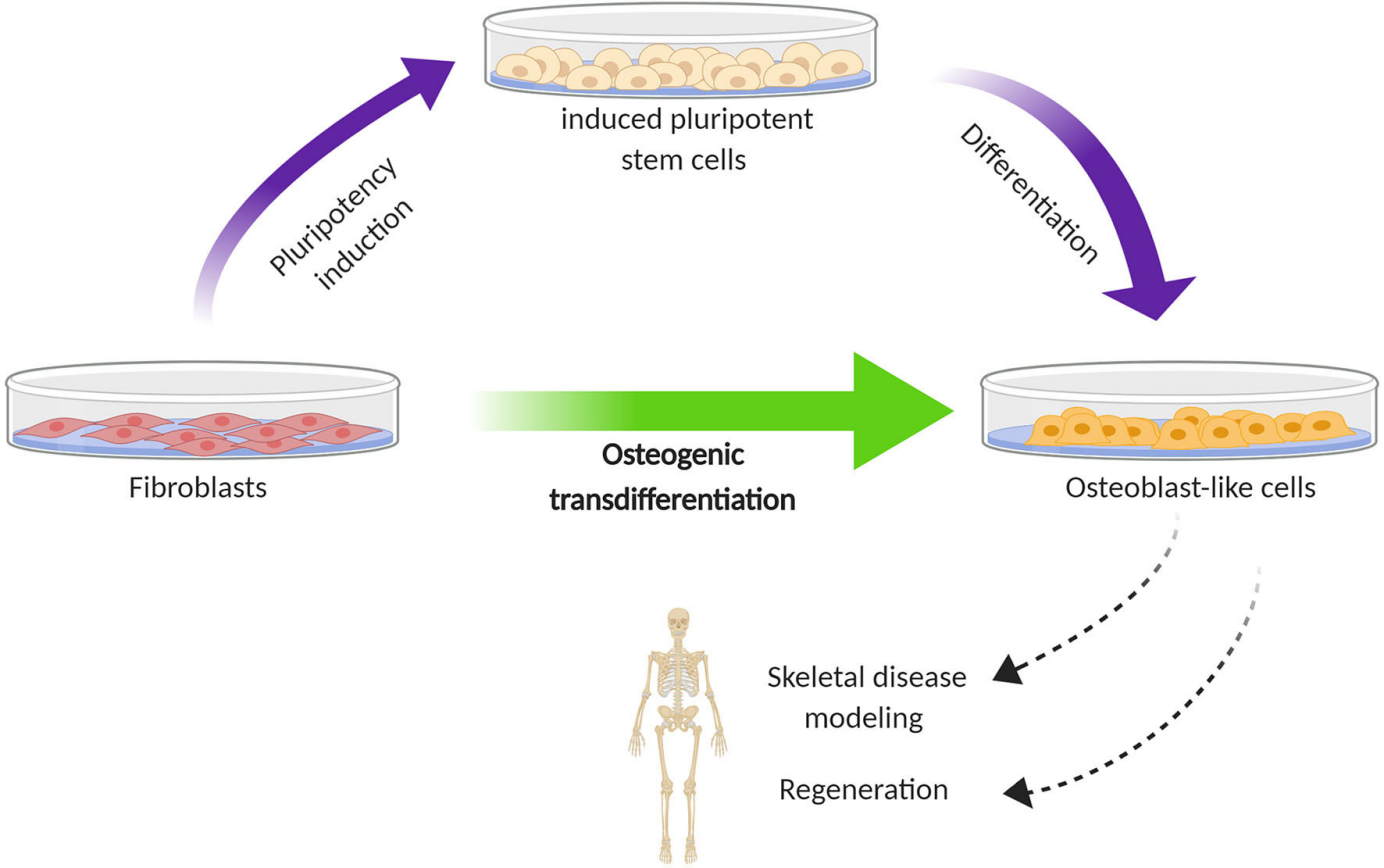
Schematic diagram illustrating the difference in cell reprogramming between iPSC-mediated differentiation (two step) and transdifferentiation (one step) for the generation of osteoblast-like cells from human fibroblasts. The first is based on directing fibroblasts toward induced pluripotent stem cells, which are subsequently subjected to osteogenic differentiation. In the second, this pluripotency stage is bypassed; fibroblasts are directly converted to osteoblast-like cells. The generation of osteoblast-like cells from fibroblasts holds promise for modeling the process of skeletal disorders and exploring regenerative therapies.

In an attempt to overcome these issues, research focus has shifted toward differentiation methods that can bypass the cumbersome step of iPSC generation (Figure 1). Transdifferentiation is the direct conversion of one differentiated cell type to another without the intermediate generation of iPSCs; however, whether and to which extent the pluripotency state is lacking, remains a point of discussion (48, 49). In addition to avoiding genomic instability and the risk of oncogenesis, an important advantage of transdifferentiation is primarily the lack of extensive cell manipulation, which means that the cells are possibly more likely to maintain their genetic makeup that may play a role in the accurate investigation of the disease mechanism. Below, several approaches of human osteoblast transdifferentiation in the field of bone disorders are summarized.

## Transgene-Mediated Osteogenic Transdifferentiation

Yamamoto et al. showed that the retroviral transduction of human gingival fibroblasts with the two osteoblast-differentiation regulators RUNX-2 and Osterix, and the two reprogramming factors Oct3/4 and L-Myc, could induce their direct conversion to osteoblast-like cells (6). The differentiated cells showed a high expression of osteoblast-related genes, produced a high amount of calcified ECM, shared a similar global gene expression pattern with primary osteoblasts, and they were able to regenerate bone defect lesions that were surgically created in the femurs of NOD/SCID mice. They differed in the lower CpG methylation at the osteocalcin gene upstream region, compared to primary osteoblasts, but which was higher compared to their progenitor fibroblast cell line. Induction of the osteoblast generation could also be achieved by transient expression of the aforementioned factors. In a similar study, the retroviral-mediated expression of combined Oct9 with N-Myc was identified as the most potent for the osteogenic conversion, which was also based on the expression of osteogenic genes RUNX-2 and osteocalcin, as well as on the production of calcified bone matrix (7). In order to avoid the unwanted effects of retroviral integration in the genome, the same group attempted the expression of Oct4, Osterix, and L-Myc with a plasmid vector in human fibroblasts (8). This led to the induction of an osteoblast-like phenotype based on the expression of osteoblast-specific genes, *in vitro* deposition of minerals, alkaline phosphatase activity, and calcified body formation following implantation in the testis of NOD/SCID mice. Regarding the latter, no teratoma formation was observed in sharp contrast to implanted iPSCs. A pertinent question is the requirement of pluripotency factors in combination with the expression of master switch genes or the common osteogenic media. Even though pluripotency was not detected in the transitioning cells, it can be assumed that they provide some level of stemness, which can prime them for osteogenic conversion by the cues provided from the other factors (6).

Considering that the TGF-β superfamily regulates diverse aspects of the skeletal system (50), the osteo-inductive properties of bone morphogenetic proteins (BMPs) have been explored. Krebsbach et al. reported that *ex vivo* adenovirus BMP-7-transduced fibroblasts have bone-forming properties when transplanted into immunocompromised mice (9). The same group subsequently showed that adenovirus BMP-7-transduced fibroblasts via subcutaneous injection can form ossicles in mice and they can also repair segmental defects in rat femurs. The *in vivo* osteoblast conversion of the transduced fibroblasts in the diffusion chambers took place without contact with the host cells, stressing the osteogenic role of BMP-7 (10). This is in agreement with the suppression of the osteoblast phenotype after addition of the BMP inhibitor noggin in osteogenic media of human fibroblasts seeded in p-tricalcium phosphate scaffolds (19). Chen et al. also showed that the knockdown of the BMP signaling-regulator SMAD4 attenuates the osteogenic differentiation of fibroblasts after adenovirus-mediated BMP-7 expression (11). These studies have not compared BMP-7 with other BMPs, which have also shown to have osteogenic capacity in mouse fibroblasts (51); whether this applies to human fibroblasts remains to be shown.

## Transgene Free-Mediated Osteogenic Transdifferentiation

In the described studies, as well as in other studies with untransfected fibroblasts (12), cells were treated with osteogenic media, which included supplementation with ascorbic acid, β-glycerophosphate, and dexamethasone. Dexamethasone is a synthetic glucocorticoid which is frequently used in recipes of osteogenic media to promote the *in vitro* commitment of cells toward the osteogenic cell lineage (52). However, glucocorticoid-induced osteoporosis clearly points to a differential effect on osteogenesis; the boundary distinguishing its ability to promote or suppress bone formation is still undefined (53, 54). In order to provide an alternative for dexamethasone and fetal calf serum, we have turned to growth factors. We have developed an *in vitro* method of osteogenic transdifferentiation based on human platelet lysate (13, 14). Platelet lysate provides numerous growth factors (55), which have been shown to promote osteogenic differentiation of bone marrow-derived MSCs (56, 57) and prevent osteoporosis development in ovariectomized mice (57). In this model, we have observed that dermal fibroblasts from FOP patients show an enhanced potential for osteogenic transdifferentiation in agreement with the heterotopic ossification characterizing this disease (13) A similar model for osteogenesis in FOP also exists with periodontal ligament fibroblasts (15). We also used our model to investigate the effect of the identified genetic variants in *AIFM1* on protein level in patients with spondylometaphyseal dysplasia (14). *AIFM1* encodes the mitochondrial apoptosis-inducing factor 1, which was undetectable in dermal fibroblasts; the osteogenic transdifferentiation of fibroblasts to osteoblast-like cells allowed the confirmation of the pathogenic effect of the *AIFM1* variant in the differentiated cell type modeling the disease. Differentiation of fibroblasts toward the osteoblast lineage was also demonstrated with treatment of osteoclast-conditioned media (16). It is known that osteoclasts secrete factors affecting osteoblast differentiation (58); the osteoclast factors mediating the osteoblast conversion were not addressed in this study. The identification of key osteogenic factors in the platelet lysate and osteoclast-conditioned media can aid the optimization of transgene-free protocols.

The chemical inhibition of the ALK5 receptor, a TGF-β type I receptor mediating signaling by TGF-β ligands, with the use of the ALK5 inhibitor II, directed the conversion of human dermal fibroblasts to osteoblast-like cells (17). In particular, the combination of ALK5 inhibitor II and vitamin D3 yielded the highest enhancement in osteoblast differentiation. The implantation of the differentiated cells in created bone lesions of immunodeficient NOG mice resulted in bone healing, as evaluated by histological analysis of callus formation and ossification. Interestingly, the stimulation of osteoblastogenesis by ALK5 inhibition is the opposite of what we observed in our study with the different ALK5 inhibitor GW788388 (13). In another study, the addition of TGF-β to osteogenic media was shown to improve the capacity of dermal fibroblasts for osteogenic transdifferentiation, although it did not contribute to mineralization (18). These differences may be attributed to the different growth factor compositions between these models, as well as to the different properties of the chemical inhibitors; for example, GW788388 is known to additionally target ALK4 and ALK7, which are TGF-β type I receptors for activin signaling (59). In a different approach, Cho et al. used BMP2 treatment combined with compound-induced demethylation of the hypermethylated CpG islands of the *RUNX2* and *ALP* genes to drive the direct differentiation of human gingival fibroblasts to functional osteoblasts, as shown by the subcutaneous ectopic bone formation in BNX mice after implantation of the epigenetically modified cells (20). These studies highlight the potential of chemical approaches in osteogenic transdifferentiation as a more controlled, simple, and low-cost alternative to growth factors.

## Chondrogenic Transdifferentiation

Bone development takes place through two different modes: intramembranous or endochondral ossification. The first is characterized by the differentiation of progenitor mesenchymal cells to osteoblasts, whereas the second is mediated by an intermediate cartilage phase preceding bone tissue development (60). Thus, given that endochondral ossification is an integral part of skeletogenesis, the availability of models to study the chondrocytes certainly has the possibility to deliver significant insights into the dysregulation of this process in certain disorders (61). Similar to osteogenic transdifferentiation, several protocols exist for chondrogenic transdifferentiation of fibroblasts, which are based on growth factor stimulation, forced expression of key transcription factors, scaffold biomaterials, and hypoxic conditions (62).

## Conclusions

Osteogenic transdifferentiation is an attractive route to generate cells of the osteogenic cell lineage. Available examples show that they can be promising in modeling of bone diseases. Several studies exist presenting different experimental options for fibroblast commitment toward the osteogenic cells; many of these do not make use of transgene introduction, which offers an advantage over iPSCs. The latter are derived after fibroblast reprogramming and subsequent differentiation, a process that requires extensive genetic modification, is technically demanding, and may pose malignancy risks in tissue regeneration. On the other hand, the fact that limited studies exist about osteogenic transdifferentiation means that we still have an incomplete understanding of the mechanism and the whole spectrum of potential advantages and shortcomings. With this review, we hope to generate excitement and ideas about the under-investigated osteogenic transdifferentiation as an alternative for the iPSC detour. Harnessing the osteogenic potential of the easily attainable fibroblasts is an attractive prospect for the study of bone pathophysiology and the future development of new technologies for bone regeneration therapy.

## Author Contributions

DM has carried out the conception and writing of the article. All authors contributed to the article and approved the submitted version.

## Conflict of Interest

The authors declare that the research was conducted in the absence of any commercial or financial relationships that could be construed as a potential conflict of interest.
